# Long non‐coding RNA MYOSLID functions as a competing endogenous RNA to regulate MCL‐1 expression by sponging miR‐29c‐3p in gastric cancer

**DOI:** 10.1111/cpr.12678

**Published:** 2019-09-09

**Authors:** Yuying Han, Nan Wu, Mingzuo Jiang, Yi Chu, Zhiyang Wang, Hao Liu, Jiayi Cao, Hanming Liu, Bing Xu, Xin Xie

**Affiliations:** ^1^ Laboratory of Tissue Engineering, Faculty of Life Science Northwest University Xi’an China; ^2^ State key Laboratory of Cancer Biology, National Clinical Research Center for Digestive Diseases and Xijing Hospital of Digestive Diseases Air Force Military Medical University Xi’an China; ^3^ College of Computer Science and Technology Jilin University Changchun China; ^4^ Department of Gastroenterology Second Affiliated Hospital of Xi’an Jiaotong University Xi’an China

**Keywords:** ceRNA, Gastric cancer, lncRNA MYOSLID, miR‐29c‐3p, myeloid cell leukaemia‐1 (MCL‐1)

## Abstract

**Objective:**

Long non‐coding RNA (lncRNA) has become an important regulator of many human malignancies. However, the biological role and clinical significance of most lncRNA in gastric cancer (GC) remain unclear.

**Methods:**

We investigate the biological function, mechanism of action and clinical expression of lncRNA MYOSLID in GC. First, we analysed the differential expression of lncRNA MYOSLID in GC tissues and non‐cancerous tissues by analysing the sequencing data obtained from The Cancer Genome Atlas. Subsequently, we verified that lncRNA MYOSLID regulates the proliferation and apoptosis of GC cells by acting as a ceRNA against miR‐29c‐3p. The nude mouse xenograft was used to further confirm the functional significance of lncRNA MYOSLID in vivo.

**Results:**

We found for the first time that the expression of lncRNA MYOSLID was significantly up‐regulated in GC tissues, and the up‐regulation of lncRNA MYOSLID in GC was correlated with tumour size, AJCC stage, depth of invasion and survival time. In addition, apoptosis and growth arrest can be induced in vitro after knockdown of lncRNA MYOSLID, which inhibits tumorigenesis in mouse xenografts in vivo. Further in‐depth studies revealed that lncRNA MYOSLID acts as a ceRNA of miR‐29c‐3p, resulting in de‐repression of its downstream target gene MCL‐1.

**Conclusion:**

The lncRNA MYOSLID‐miR‐29c‐3p‐MCL‐1 axis plays a key role in the development of GC. Our findings may provide potential new targets for the diagnosis and treatment of human GC.

## 1 INTRODUCTION

Gastric cancer (GC) is the leading cause of cancer‐related death worldwide due to its high morbidity and lack of effective treatments.^1^ Despite advances in surgical techniques, molecular‐targeted therapies and oncology immunotherapy, the overall 5‐year survival rate for patients with GC remains low.^2^, ^3^ Since there are few specific symptoms in the early stage of GC, most patients have already had lymph node metastasis at the time of diagnosis.^4^ At present, there is no clear molecular feature in the diagnosis and treatment of GC. Therefore, it is important to find biomarkers and new effective therapeutic targets for the diagnosis and treatment of GC.

Long non‐coding RNAs (lncRNAs) are a class of transcripts greater than 200 nucleotides in length with limited protein‐coding ability or no protein‐coding ability.^5^ It has been found that lncRNA is abundantly transcribed in mammalian cells and in plant cells.^6^, ^7^ These lncRNAs can be involved in a number of important cellular biological processes, including regulation of cell growth,^8^ apoptosis,^9^ cell differentiation,^10^ and cell invasion and metastasis.^11^ Increasing evidence showed that lncRNA can be used as a biomarker for diagnosis and prognosis in a variety of cancers, such as colorectal cancer,^12^ breast cancer,^13^ liver cancer,^14^ prostate cancer^15^ and GC.^16^ Typically, lncRNAs exert their biological functions by regulating epigenetic,^17^ transcriptional^18^ and post‐transcriptional levels^19^ that regulate potential target gene expression. In recent years, more and more studies have shown that lncRNA plays an important role in human cancer.^6^ For example, LINC00941 is significantly up‐regulated in liver cancer and is significantly associated with poor clinical outcomes, and regulates the metastasis and proliferation of liver cancer by binding ANXA2 to affect the activity of the Wnt/β‐catenin signalling pathway.^20^ Furthermore, in colorectal cancer, lncRNA UICLM inhibits the expression of miR‐150‐5p by competitive endogenous RNA action, thereby promoting liver metastasis of colorectal cancer.^21^ It is well documented that a number of important lncRNAs have been proved to be significant survival prognosis of GC. For example, lncRNA HOTAIR promotes gastric cancer metastasis by binding to the epigenetic transcriptional regulator polycomb inhibitor complex 2 (PRC2).^21^ HOTAIR also regulates cisplatin resistance in GC by acting as a competitive endogenous RNA (ceRNA) of miR‐126.^22^ In addition, LINC01234 functions as a competing endogenous RNA to regulate CBFB expression by sponging miR‐204‐5p regulates the malignant proliferation of GC.^4^

LncRNA MYOSLID was first reported in human VSMC‐selective and serum‐responsive factor/CArG‐dependent lncRNA, which regulates VSMC differentiation through the MKL1 and transforming growth factor‐beta/SMAD pathways.^23^ We have previously studied the differentially expressed lncRNA in GC from the Cancer RNA‐Seq Nexus database and found that the expression of lncRNA MYOSLID in GC is significantly different and is associated with the survival prognosis of GC.^24^ However, the mechanism of lncRNA MYOSLID in GC remains elusive.

In this study, we studied lncRNA MYOSLID in GC. We first discovered that lncRNA MYOSLID is significantly up‐regulated in GC tissues and is associated with poor prognosis. Loss and functional gain assays showed that lncRNA MYOSLID promotes GC cell proliferation and inhibits apoptosis by acting as a miR‐29c‐3p ceRNA, thereby preventing miR‐29c‐3p from binding to the target protein MCL‐1. Collectively, the results suggested that lncRNA MYOSLID is an oncogenic regulator of tumorigenesis in GC and may be a potential target for the diagnosis and treatment of patients with GC.

## 2 MATERIALS AND METHODS

### 2.1 Tissue samples

Seventy‐five patients with gastric adenocarcinoma and paired normal tissues were obtained from patients undergoing GC surgery at Xijing Digestive Disease Hospital. All samples were clinically and pathologically validated. This study was approved by the Xijing Hospital Human Body Protection Committee. Informed consent was obtained from each patient.

### 2.2 Cell culture

Human GC cell lines MKN45, AGS, SGC‐7901 and BGC‐823 were purchased from Institute of Biochemistry and Cell Biology of the Chinese Academy of Sciences (Shanghai, China). The immortal normal gastric epithelial cell line GES‐1 was purchased from the Institute of Biochemistry and Cell Biology of the Chinese Academy of Sciences (Shanghai, China). All cells were cultured in DMEM basic containing 10% foetal bovine serum (Gibco) and 1% penicillin‐streptomycin (Gibco). All cells were incubated with 5% (v/v) CO^2^ at 37°C. All cells were tested for mycoplasma contamination before the experiments.

### 2.3 RNA extraction, reverse transcription and real‐time RT‐PCR

Total RNA from cells was extracted using an RNA isolation kit (TaKaRa, Tokyo, Japan) according to the manufacturer's instructions. Subsequently, the RevertAid First Strand cDNA Synthesis Kit (TaKaRa, Tokyo, Japan) was used to reverse‐transcribe the messenger RNA (mRNA) from the total mRNA; primers for miR‐29c‐3p and U6 were purchased from RiboBio (Guangzhou, China); the specific primer (Table S2) and the SYBR premix Ex Taq (TaKaRa, Tokyo, Japan) were used to expand by real‐time qPCR (Bio‐Rad, CA, USA). It was carried out with the following parameters: pre‐denaturation at 95°C for 5 minutes, denaturation at 95°C for 10 seconds, annealing at 62°C for 20 seconds and extension at 72°C for 30 seconds for 40 cycles. Glyceraldehyde‐3‐phosphate dehydrogenase (GAPDH) was used as an internal control.

### 2.4 Western blot analysis

The cells were washed three times with PBS and collected in RIPA lysis buffer (Beyotime Biotechnology, Shanghai, China) supplemented with a protease inhibitor cocktail (Calbiochem, San Diego, USA). Protein concentration was determined by staining with Coomassie Blue (Beyotime Biotechnology, Shanghai, China). Cellular protein lysates were separated by 10% sodium dodecyl sulphate‐polyacrylamide gel electrophoresis (SDS‐PAGE), transferred to a 0.22 mm polyvinylidene fluoride membrane (Millipore) and probed with specific antibodies. Specific bands were detected by ECL chromogenic substrate and quantified by densitometry (Quantity One software, Bio‐Rad). The GAPDH antibody was used as a control. Anti–caspase‐3, cleaved caspase‐3, poly (ADP ribose) polymerase protein (PARP), cleaved PARP, cyclin D1, CDK2 and MCL‐1 (1:1000) were purchased from Cell Signalling Technology. GAPDH antibody was purchased from Proteintech. All antibodies are listed in Table S3.

### 2.5 *RNA fluorescent *in situ* hybridization*

The subcellular localization of lncRNA MYOSLID was detected by FISH kit (RiboBio, Guangzhou, China) according to the manufacturer's instructions. The Cy3 labelled lncRNA MYOSLID probe was obtained from RiboBio (Guangzhou, China). Briefly, gastric cancer cells (2 × 10^4^) were seeded on cell slides in 24‐well culture plates. After waiting for the cells to adhere, the cells were fixed in 4% paraformaldehyde for 30 minutes at room temperature. After permeabilization, the cells are pre‐hybridized with the pre‐hybridization solution and the hybridization solution and then incubated with the cy3‐labelled lncRNA MYOSLID oligonucleotide probe. The nuclei were stained with DAPI for 10 minutes at room temperature.

### 2.6 RNA immunoprecipitation

RNA immunoprecipitation was performed using the EZ‐Magna RIP kit (Millipore, Billerica, MA, USA) according to the manufacturer's instructions. First, we lysed SGC‐7901 and BGC‐823 cells and incubated with Protein A magnetic beads; next, we conjugated the magnetic beads to the antibody at 4°C. After 3‐6 hours, the beads were washed with washing buffer and then incubated with 0.1% SDS/0.5 mg/mL proteinase K for 30 minutes at 55°C to remove proteins. Finally, we performed qRT‐PCR analysis of immunoprecipitated RNA using primers specific for lncRNA MYOSLID.

### 2.7 Virus

Virus packaging was performed in HEK293T cells by co‐transfection with lentiviral vectors with the packaging plasmid pHelper 1.0 vector (GeneChem Co., Ltd., Shanghai, China) and the envelope plasmid pHelper 2.0 vector (GeneChem Co., Ltd.) using Lipofectamine 2000 (Invitrogen). At 48 hours after transfection, supernatants containing lentiviral particles were collected, and the virus titre was quantified according to the manufacturer's instructions. Lentiviral vectors encoding short hairpin RNAs (shRNAs) (Sh: ATTATTGTAACCACCCGTT) targeting MYOSLID were generated using the GV344 vector (hU6‐MCS‐Ubiquitin‐Luc_firefly IRES‐puromycin, GeneChem Co., Ltd., Shanghai, China). A scrambled GV344 vector (TTCTCCGAACGTGTCACGT) was used as the negative control. Stable transfectants overexpressing MYOSLID were generated by lentiviral transduction using a GV341 vector (Ubi‐MCS‐3FLAG‐SV40‐puromycin, GeneChem Co., Ltd.). An empty vector was used as the negative control.

### 2.8 Animal experiments

Control shRNA‐Ctrl or sh‐MYOSLID (3 × 10^6^) stably transfected SGC‐7901 cells and carried miR‐29c‐3p, miR‐NC (negative control), sh‐MCL‐1 and empty vector (negative control)‐stained cells SGC‐7901 were injected subcutaneously into either side of the axillary region of male BALB/c nude mice (4‐5 weeks old). At 28 days after the injection, the mice were euthanized and the subcutaneous growth of each tumour was examined. This study was conducted in strict accordance with the recommendations of the National Institutes of Health Laboratory Animal Care and Use Guidelines. All experimental procedures were approved by the Xijing Hospital Institutional Review Board. Animal experiments were conducted with the approval of the Animal Research Institutions Committee and are consistent with the National Laboratory Animal Care and Use Guidelines.

### 2.9 Statistical analysis

We performed statistical analysis using Prism 5 (San Diego, CA, USA) and SPSS 18.0 (Chicago, IL, USA) software. Differences between the two groups were assessed using Student's *t* test. A *P* value < .05 was considered to represent a significant difference. The overall survival curve was estimated by the Kaplan‐Meier method and the Cox proportional hazard model. All values are expressed as mean ± SD unless otherwise stated.

## 3 RESULTS

### 3.1 lncRNA MYOSLID is up‐regulated in GC and associated with poor prognosis

**Figure 1:**
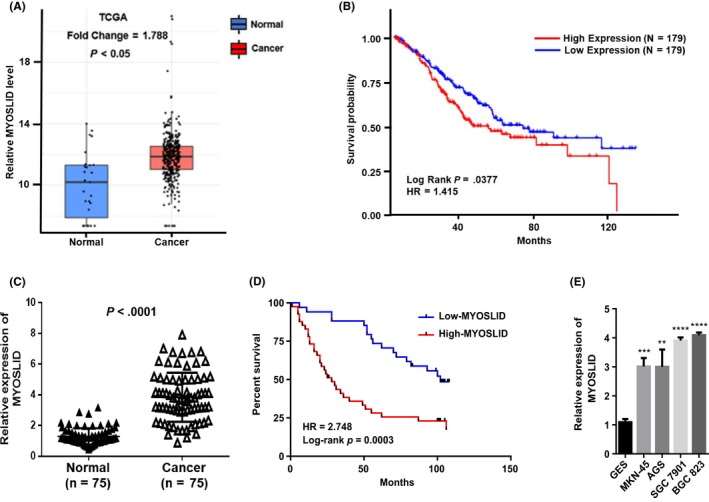
lncRNA MYOSLID is up‐regulated in gastric cancer and associated with poor prognosis. A, The relative expression of lncRNA MYOSLID in gastric cancer tissues and normal tissues was analysed using TCGA data. B, The Kaplan‐Meier curve depicts the overall survival of 358 patients with GC. C, Real‐time quantitative PCR was used to detect lncRNA MYOSLID expression in gastric cancer tissues and adjacent non‐tumour tissues (n = 75). D, Based on the expression level of lncRNA MYOSLID, the Kaplan‐Meier curve depicts the overall survival curve of 75 patients with gastric cancer. E, Real‐time quantitative PCR was used to analyse the expression of lncRNA MYOSLID in normal gastric epithelial cell line (GES‐1) and gastric cancer cells. Error bars indicate mean ± standard errors of the mean. **P < .01, ***P < .001

To investigate the expression of lncRNA MYOSLID in human GC, we searched the Cancer Genome Atlas (TCGA) database and found that the lncRNA MYOSLID gene copy number was significantly elevated in GC tissues compared with normal gastric tissue (Figure 1A). We next analysed publicly available data and found that lncRNA MYOSLID expression is closely related to the overall survival of patients with GC (Figure 1B). Then, the expression of lncRNA MYOSLID in GC tissues was detected by real‐time PCR and found that the expression of lncRNA MYOSLID was higher in GC tissues than in matched non‐tumour tissues (n = 75, *P* < .0001, Figure 1C). To assess the clinical significance of lncRNA MYOSLID overexpression in GC, we evaluated the association between lncRNA MYOSLID levels and clinicopathological features. As shown in Table S1, high lncRNA MYOSLID expression was associated with patients age (*P* = .018), larger tumour size (*P* = .001), invasion‐related depth (*P* = .010) and AJCC staging (*P* = .001), while lncRNA MYOSLID was no significant correlation between expression and other factors including gender (*P* = 1.000). We also examined the association between lncRNA MYOSLID expression levels and prognosis in patients with GC. Kaplan‐Meier survival analysis showed that patients with higher lncRNA MYOSLID levels had shorter overall survival than patients with lower lncRNA MYOSLID levels (Figure 1D). In addition, lncRNA MYOSLID was significantly overexpressed in GC cell lines compared with human normal gastric epithelial cells (GES‐1) (Figure 1E). These data suggested that IncRNA MYOSLID is involved in the pathogenesis of GC.

### 3.2 lncRNA MYOSLID silencing inhibits GC cell proliferation

**Figure 2:**
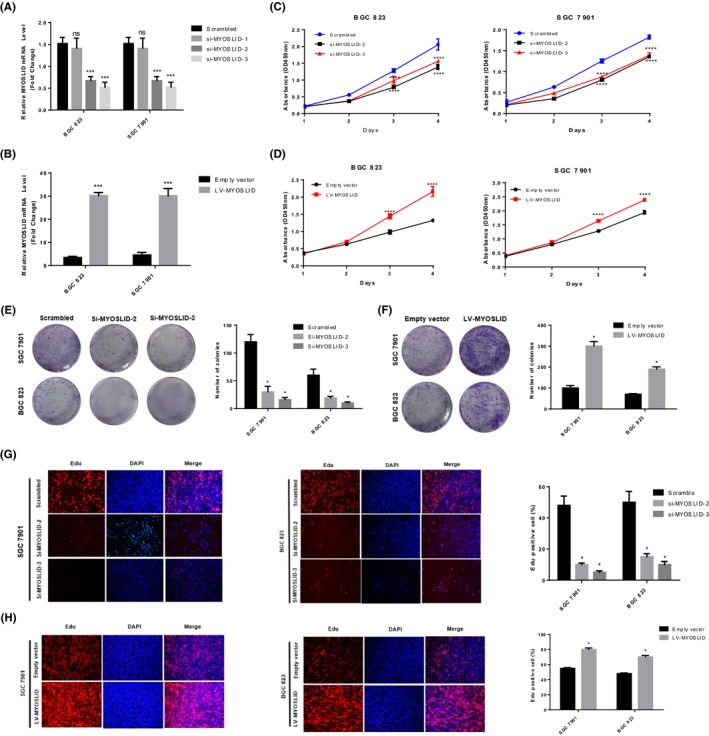
lncRNA MYOSLID silencing inhibits gastric cancer cell proliferation. A, Real‐time quantitative PCR analysis of lncRNA MYOSLID expression in scrambled, si‐MYOSLID 1#, si‐MYOSLID 2# and si‐MYOSLID 3#. B, Real‐time quantitative PCR analysis of lncRNA MYOSLID expression in empty vector and LV‐MYOSLID. C, D, The CCK8 assay was used to determine the viability of si‐MYOSLID transfected or LV‐MYOSLID transfected gastric cancer cells. E, F, Colony formation assays were performed to determine the proliferation of si‐MYOSLID‐transfected or LV‐MYOSLID–transfected gastric cancer cells. G, H, EdU staining assays were performed to determine the proliferation of si‐MYOSLID–transfected or LV‐MYOSLID–transfected gastric cancer cells. The data represent the mean ± SEM from three independent experiments. *P < .05, **P < .01, ***P < .001

To elucidate the biological function of lncRNA MYOSLID in GC cell lines, we transfected with siRNA, lentiviral recombinant short hairpin RNA (shRNA) vector or lentiviral recombinant overexpression plasmid, to knock down or overexpress lncRNA MYOSLID in GC cell lines SGC‐7901 and BGC‐823 (Figure 2A,B). CCK‐8 proliferation assay showed that lncRNA MYOSLID knockdown significantly inhibited the proliferation of SGC‐7901 and BGC‐823 cells, while lncRNA MYOSLID overexpression significantly promoted the growth of SGC‐7901 and BGC‐823 cells (Figure 2C,D). Consistently, cell colony formation assay showed that down‐regulation of lncRNA MYOSLID significantly reduced the colony formation ability of SGC‐7901 and BGC‐823 cells, but lncRNA MYOSLID overexpression significantly increased colony formation ability (Figure 2E,F). In addition, the use of EdU proliferation assay showed that knockdown of lncRNA MYOSLID significantly reduced the EdU‐positive rate of GC cells, but overexpression of lncRNA MYOSLID significantly increased the EdU‐positive rate (Figure 2G,H). These results indicated that lncRNA MYOSLID acts as an oncogene to promote malignant proliferation of GC.

### 3.3 Knockdown of lncRNA MYOSLID induces apoptosis and G1 arrest in gastric cancer cells

**Figure 3:**
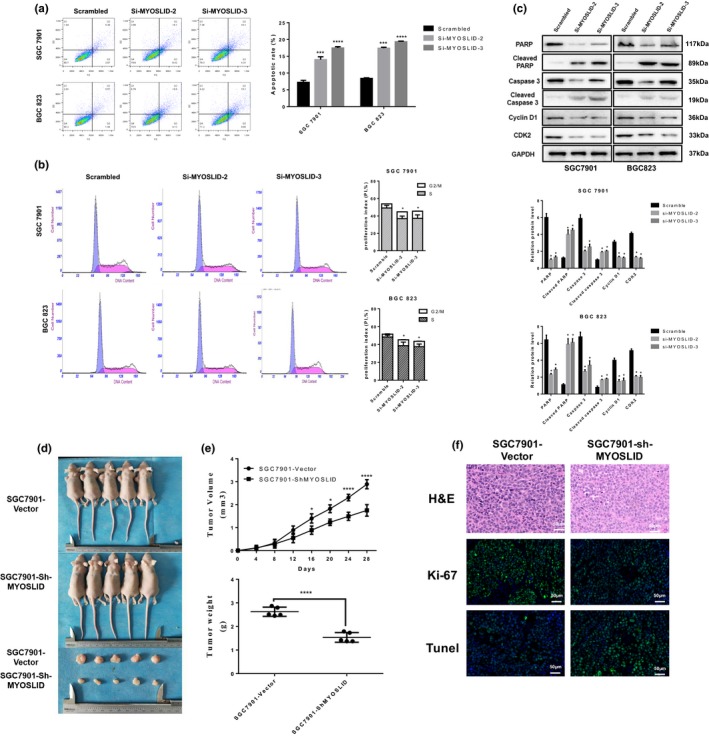
Knockdown of lncRNA MYOSLID induces apoptosis and G1 arrest in *vitro* and *vivo*. A, Flow cytometry was used to detect apoptosis rates. B, The lncRNA MYOSLID was silenced in SGC‐7901 and BGC‐823 cells, and flow cytometry showed a significant increase in the proportion of cells in the G1 phase. C, Western blot analysis of apoptosis‐related proteins and cell cycle‐associated proteins after transfection of scrambled siRNA, si‐MYOSLID 2# or si‐MYOSLID 3# in SGC‐7901 and BGC‐823 cells. The GAPGH protein was used as an internal control. Error bars indicate mean ± standard errors of the mean. D, Stable lncRNA MYOSLID knockdown SGC‐7901 cells were used for in vivo assays. Tumours from two groups of nude mice were shown and measured and showed tumour growth curves after injection of SGC‐7901 cells. Tumour volume was calculated every 4 days. E, Represents tumour weight from both groups (n = 5). F, Ki67 protein levels and apoptotic cells in tumour tissues from sh‐MYOSLID or negative control SGC‐7901 cells were determined by Immunofluorescence and TUNEL staining. The data represent the mean ± SEM from three independent experiments. *P < .05, **P < .01, ***P < .001

Malignant proliferation is one of the main causes of high mortality in GC. Increased apoptosis and cell cycle arrest are the two major factors leading to cancer cell proliferation. Therefore, we performed flow cytometry to analyse the effect of knockdown lncRNA MYOSLID on these characteristics. We found that GC cell lines SGC‐7901 and BGC‐823 transfected with lncRNA MYOSLID‐specific siRNA (si‐MYOSLID 2# or 3#) had higher apoptotic rates than cells of scrambled siRNA (Figure 3A). Meanwhile, SGC‐7901 and BGC‐823 cells transfected with lncRNA MYOSLID‐specific siRNA showed significant cell cycle arrest compared to cells transfected with scrambled siRNA (Figure 3B). In addition, significantly higher levels of apoptosis‐related proteins are expressed in cells transfected with lncRNA MYOSLID‐specific siRNA, including cleaved caspase‐3, cleaved PARP, etc At the same time, cell cycle arrest is significantly associated with G1/S checkpoint protein expression, including cyclin D1, CDK2, cyclin D3 and CDK4. We found that knockdown of lncRNA MYOSLID in GC cell lines SGC‐7901 and BGC‐823 significantly increased the expression of cleaved caspase‐3 and cleaved PARP (Figure 3C). In addition, the expression of cycle‐associated proteins (such as Cyclin D1 and CDK2) was also significantly reduced. These data indicated that inhibition of GC cell proliferation after knockdown of lncRNA MYOSLID is attributable to increased apoptosis and cell cycle arrest at the G1/S checkpoint.

### 3.4 *Knockdown of lncRNA MYOSLID inhibits gastric cancer cell tumorigenesis *in vivo

To determine whether lncRNA MYOSLID affects tumour growth in vivo, the GC cell line SGC‐7901 was stably transfected with a control vector or shRNA targeting lncRNA MYOSLID and inoculated subcutaneously into female nude mice. All mice developed tumours at the injection site. We found that the size and weight of tumours in knockdown lncRNA MYOSLID group were significantly decreased compared with the empty vector group. (Figure 3D,E). Immunofluorescence of Ki‐67 and terminal deoxynucleotidyl transferase‐mediated dUTP‐fluorescein nick end labeling (TUNEL) staining in xenograft tissues showed knockdown lncRNA MYOSLID decreased the proportion of Ki‐67–positive cells and increased the proportion of apoptotic cells (Figure 3F). Taken together, these results confirmed the carcinogenic activity of lncRNA MYOSLID in GC in vivo.

### 3.5 LncRNA MYOSLID acts as a molecular sponge for miR‐29c‐3p in GC cells

**Figure 4:**
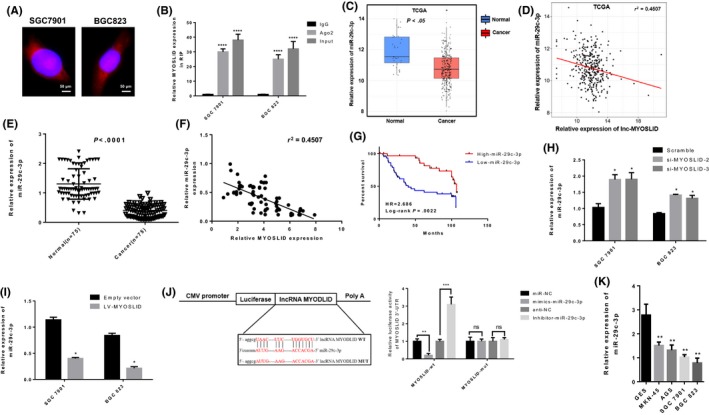
LncRNA MYOSLID acts as a molecular sponge for miR‐29c‐3p in gastric cancer cells. A, In situ hybridization experiments were performed to analyse the location of lncRNA MYOSLID (red) in the cytoplasmic and nuclear portions (blue) of SGC‐7901 and BGC‐823 cells. B, RIP experiments were performed in SGC‐7901 and BGC‐823 cells, and coprecipitated RNA was subjected to qRT‐PCR for lncRNA MYOSLID. The expression level of lncRNA MYOSLID in Ago2 RIP was significantly increased compared with its matched IgG control. C, The relative expression of miR‐29c‐3p in normal tissues of gastric cancer was analysed using the TCGA dataset. D, Correlation analysis of the relationship between lncRNA MYOSLID and miR‐29c‐3p expression levels in the TCGA data set. E, Real‐time quantitative PCR was used to detect miR‐29c‐3p expression in gastric cancer tissues and adjacent non‐tumour tissues (n = 75). F, Correlation analysis of the expression of lncRNA MYOSLID and miR‐29c‐3p in 75 cases of gastric cancer. G, Based on the expression level of miR‐29c‐3p, the Kaplan‐Meier curve depicts the overall survival curve of 75 patients with gastric cancer. H, I, After transfecting scrambled siRNA, lncRNA MYOSLID siRNA or LV‐MYOSLID in SGC‐7901 and BGC‐823 cells, the expression level of miR‐29c‐3p was detected by real‐time quantitative PCR. J, A luciferase reporter plasmid containing wild‐type (WT) or mutant (Mut) MYOSLID was co‐transfected into HEK‐293T cells in parallel with an empty plasmid vector using miR‐29c‐3p. K, Real‐time quantitative PCR was used to analyse the expression of miR‐29c‐3p in normal gastric epithelial cell line (GES‐1) and gastric cancer cells. Value represents the mean ± SEM of three independent experiments. *P < .05, **P < .01, ***P < .01

Previous studies have shown that the main forms of action of lncRNAs include RNA‐binding protein interactions or miRNAs as miRNAs to regulate expression of downstream target genes. To explore the potential molecular mechanisms of lncRNA MYOSLID in GC cell proliferation, we used in situ hybridization to analyse the subcellular localization of lncRNA MYOSLID for the first time. The results showed that the expression of lncRNA MYOSLID was mainly concentrated in the cytoplasm (Figure 4A), suggesting that the function of lncRNA MYOSLID may be post‐transcriptional level regulation of target expression. Then, we used Ago2 antibody in GC cells SGC‐7901 and BGC‐823 for RNA‐binding protein immunoprecipitation to elucidate that lncRNA MYOSLID binds directly to Ago2, which is one of the important components of RNA‐induced silencing complexes, mainly involved in miRNA‐mediated mRNA inhibition (Figure 4B). From these results, we initially hypothesized that the molecular mechanism of lncRNA MYOSLID in GC cells may act as a ceRNA of miRNA. To further confirm this hypothesis, we used an online bioinformatics database (Cancer RNA‐Seq Nexus database) to analyse predicted miRNAs that bind to the lncRNA MYOSLID sequence. The data indicate the presence of a putative binding site between lncRNA MYOSLID and miR‐29c‐3p. Then, we analysed RNA sequencing data from GC tissues from TCGA and found that miR‐29c‐3p was significantly down‐regulated in gastric tissue (Figure 4C). Additionally, we analysed the expression levels of lncRNA MYOSLID and miR‐29c‐3p in GC tissue RNA sequencing data from TCGA and found a negative correlation (Figure 4D). At the same time, we analysed the expression of miR‐29c‐3p in 75 pairs of GC tissues by qRT‐PCR and found that the expression level of miR‐29c‐3p in cancer tissues was significantly lower than that in adjacent tissues (Figure 4E). In addition, qRT‐PCR analysis of lncRNA MYOSLID and miR‐29c‐3p in 75 gastric cancer tissues revealed a significant negative correlation between the expression levels of lncRNA MYOSLID and miR‐29c‐3p (Figure 4F). Meanwhile, the Kaplan‐Meier survival analysis showed that patients with higher expression levels of miR‐29c‐3p had longer overall survival than patients with low expression of miR‐29c‐3p (Figure 4G). Then, we detected the expression level of miR‐29c‐3p in SGC‐7901 and BGC‐823 cells after knockdown or overexpressing lncRNA MYOSLID. Interestingly, the knockdown of lncRNA MYOSLID significantly increased the expression level of miR‐29‐3p (Figure 4H). Meanwhile, overexpression of lncRNA MYOSLID significantly inhibited the expression level of miR‐29c‐3p (Figure 4I). Then, we determined the change in the activity of lncRNA MYOSLID by the luciferase reporter gene after site‐directed mutagenesis by the putative miR‐29c‐3p binding site in the lncRNA MYOSLID sequence. As expected, the luciferase reporter assay showed that miR‐29c‐3p directly targets the 3'UTR of lncRNA MYOSLID‐WT to negatively regulate the luciferase activity of lncRNA MYOSLID‐wt‐3'UTR, rather than lncRNA MYOSLID‐MUT's 3'UTR (Figure 4J). As shown in Figure 4K, the expression level of miR‐29c‐3p in GC cell lines was significantly lower than that in normal gastric epithelial cells.

### 3.6 The biological function of lncRNA MYOSLID is partly mediated by the negative regulation of miR‐29c‐3p

**Figure 5:**
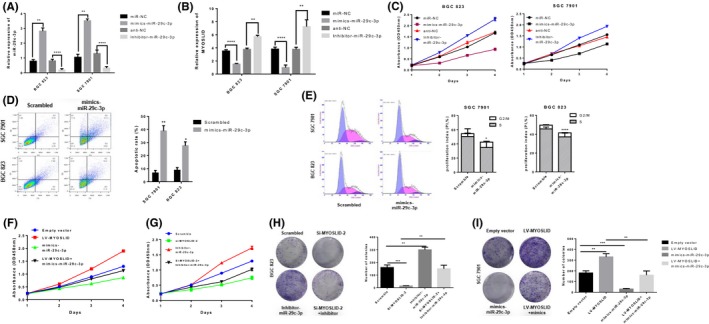
Effects of miR‐29c‐3p on gastric cancer cells proliferation, cell cycle and apoptosis in vitro. A, MiR‐29c‐3p expression was detected in SGC‐7901 and BGC‐823 cells by qRT‐PCR after transfection of miR‐29c‐3p mimic, miR‐29c‐3p inhibitor or control miRNA. B, After transfection of the miR‐29c‐3p mimetic, miR‐29c‐3p inhibitor or control miRNA, lncRNA MYOSLID expression was detected in SGC‐7901 and BGC‐823 cells by qRT‐PCR. C, After transfection of miR‐29c‐3p mimic, miR‐29c‐3p inhibitor or control miRNA, the proliferation of SGC‐7901 and BGC‐823 cells was detected by CCK‐8. D, Flow cytometry apoptosis assay was used to analyse apoptosis in BGC‐823 and SGC‐7901 cells transfected with miR‐29c‐3p mimics. E, Flow cytometry assays were performed to analyse cell cycle progression when miK‐29c‐3p was used to mimic transfected SGC‐7901 and BGC‐823 cells. F, The growth curve of SGC7901 cells was co‐transfected with LV‐MYOSLID, miR‐29c‐3p mimics or scrambled siRNA by CCK8. **(G).** The growth curve of BGC‐823 cells was co‐transfected with si‐MYOSLID 2#, miR‐29c‐3p inhibitor or scrambled siRNA by CCK8. H, The colony‐forming ability of BGC‐823 cells was co‐transfected with si‐MYOSLID 2#, miR‐29c‐3p inhibitor or scrambled siRNA by colony formation assay. I, The colony‐forming ability of SGC‐7901 cells after co‐transfection with LV‐MYOSLID, miR‐29c‐3p mimics or scrambled siRNA was determined by colony formation assay. Values represent the mean ± SEM of three independent experiments. *P < .05, **P < .01, **P < .001

To determine the role of miR‐29c‐3p in GC cells, we transfected miR‐29c‐3p mimics or miR‐29c‐3p inhibitors in GC cell lines SGC‐7901 and BGC‐823 (Figure 5A). At the same time, we found that when cells were transfected with miR‐29c‐3p mimics or miR‐29c‐3p inhibitors, lncRNA MYOSLID also produced significant changes (Figure 5B). Then, CCK‐8 and colony formation assay showed that miR‐29c‐3p overexpression significantly reduced cell proliferation and colony‐forming ability, while inhibition of miR‐29c‐3p expression significantly enhanced cell proliferation and colony‐forming ability (Figure 5C, H,I). In addition, flow cytometry analysis showed that overexpression of miR‐29c‐3p in GC cell lines SGC‐7901 and BGC‐823 significantly increased apoptotic rate and cell cycle arrest cell numbers (Figure 5D,5). Western blot analysis showed that miR‐29c‐3p mimics transfected SGC‐7901 and BGC‐823 cells significantly increased the levels of apoptosis‐related proteins including cleaved PARP and cleaved caspase‐3 protein, and significantly decreased cycle‐related proteins including cyclin D1 and CDK2 (Figure S1A). To determine that the biological function of lncRNA MYOSLID is partially mediated by the negative regulation of miR‐29c‐3p, SGC‐7901 cells were co‐transfected with si‐MYOSLID‐2 and miR‐29c‐3p inhibitors. Notably, si‐MYOSLID‐2–mediated inhibition of cell proliferation was partially rescued by co‐transfection with a miR‐29c‐3p inhibitor (Figure 5F, G). Similarly, we transfected miR‐29c‐3p mimics in BGC‐823 cells that overexpressing lncRNA MYOSLID. The results showed that the proliferation of GC cells promoted by overexpression of lncRNA MYOSLID was partially inhibited by miR‐29c‐3p mimics transfection (Figure 5H,I). All mice developed tumours at the injection site. We found that the size and weight of tumours in the overexpressed miR‐29c‐3p group were significantly reduced compared with the empty vector group (Figure S1B,C). Immunofluorescence of Ki‐67 and terminal deoxynucleotidyl transferase‐mediated dUTP‐fluorescein nick end labeling (TUNEL) staining were used in xenograft tissues. The results showed that overexpression of miR‐29c‐3p reduced Ki‐67–positive cells while increasing the proportion of apoptotic cells (Figure S1D). These results indicated that the function of lncRNA MYOSLID in GC cells is at least partly mediated by the negative regulation of miR‐29c‐3p.

### 3.7 MCL‐1 is a miR‐29c‐3p target gene and is indirectly regulated by lncRNA MYOSLID

**Figure 6:**
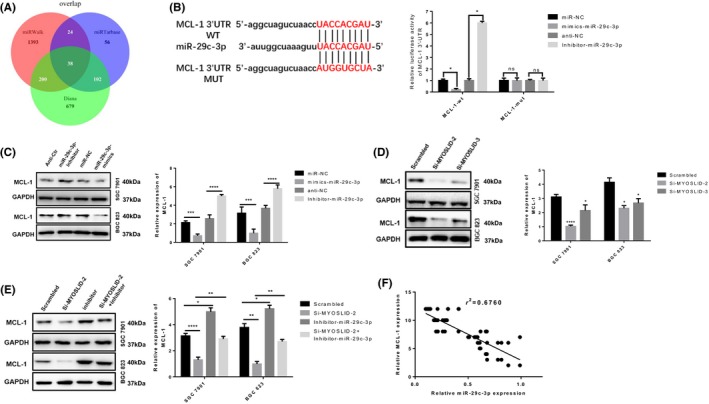
MCL‐1 is a miR‐29c‐3p target gene and is indirectly regulated by lncRNA MYOSLID. A, LncRNA MYOSLID‐miR‐29c‐3p–targeted ceRNA network. B, Schematic representation of the putative targeting site of miR‐29c‐3p in WT and Mut 3'UTR of Mcl‐1 (left). Luciferase activity assay in HEK‐293T cells transfected with a luciferase reporter plasmid containing Mcl‐3 'UTR (WT or Mut) and control miRNA or miR‐29c‐3p. C, Protein levels of MCL‐1 in SGC‐7901 and BGC‐823 cells transfected with scrambled miRNA, miR‐29c‐3p‐inhibitor or miR‐29c‐3p‐mimetic. D, MCL‐1 protein levels in GC cell lines SGC‐7901 and BGC‐823 after knockdown of lncRNA MYOSLID. E, MCL‐1 protein levels in SGC‐7901 and BGC‐823 cells after knockdown of lncRNA MYOSLID and/or inhibition of miR‐29c‐3p. F, Correlation analysis of the relationship between miR‐29c‐3p and MCL‐1 expression in gastric cancer tissues (n = 75). *P < .05, **P < .01, ***P < .001

The role of ceRNA regulatory networks in GC has been widely reported. To determine the ceRNA regulatory network between lncRNA MYOSLID, miR‐29c‐3p and downstream targets, we used a network database (miRWalk, miRtarbase and Diana) to predict potential miR‐29c‐3p target genes. In addition, we predicted the lncRNA MYOSLID‐miR‐29c‐3p targeting ceRNA network using their expression in TCGA data and found that genes such as MCL‐1, CCNA2 and DDX21 may be involved in this network (Figure 6A). MCL‐1 protein is a special protein in the process of controlling apoptosis, and MCL‐1 can protect tumour cells against apoptosis. Next, we performed a luciferase reporter gene assay driven by the wild‐type 3'UTR sequence of MCL‐1, which contains the predicted miR‐29c‐3p–binding site (wt‐MCL‐1), or mutant constructs containing a mutation in the miR‐29c‐3p–binding sites (mut‐MCL‐1). These plasmids were co‐transfected into HEK293T cells with non‐targeting control miRNA, miR‐29c‐3p mimics and miR‐29c‐3p inhibitor. The results showed that co‐transfection with miR‐29c‐3p mimic significantly reduced wt‐MCL‐1–driven luciferase expression compared with control, and co‐transfection of miR‐29c‐3p inhibitor increased wt‐MCL‐1 drives luciferase expression, but this change is abolished by mutation of the putative miR‐29c‐3p binding site (Figure 6B). To determine whether MCL‐1 is regulated by miR‐29c‐3p in GC cells, we measured the protein level of MCL‐1 when miR‐29‐3p was overexpressed or inhibited in SGC‐7901 and BGC‐823 cells. We found that overexpression or inhibition of miR‐29c‐3p significantly reduced or increased protein levels of MCL‐1, respectively (Figure 6C). Since lncRNA MYOSLID could bind to miR‐29‐3p, we next determined whether lncRNA MYOSLID could regulate MCL‐1 expression by binding to the same site in miR‐29‐3p. We found that knockdown of lncRNA MYOSLID also significantly reduced the protein level of MCL‐1 in SGC‐7901 and BGC‐823 cells (Figure 6D). To determine whether miR‐29c‐3p plays a role in the relationship between lncRNA MYOSLID and MCL‐1, we examined SGC‐7901 and BGC‐823 cells co‐transfected with si‐MYOSLID and miR‐29c‐3p inhibitors. In fact, si‐MYOSLID‐2–mediated reduction in MCL‐1 protein levels was effectively reversed by miR‐29c‐3p inhibitors (Figure 6E). In addition, we analysed the association between miR‐29c‐3p and MCL‐1 expression in 75 pairs of GC tissues and found a negative correlation between miR‐29c‐3p and MCL‐1 (Figure 6F). Taken together, these data suggest that MCL‐1 expression regulation is primarily mediated by post‐transcriptional regulation of miR‐29c‐3p via lncRNA MYOSLID.

### 3.8 MCL‐1 expression is up‐regulated in GC tissues and promotes GC cell growth

**Figure 7:**
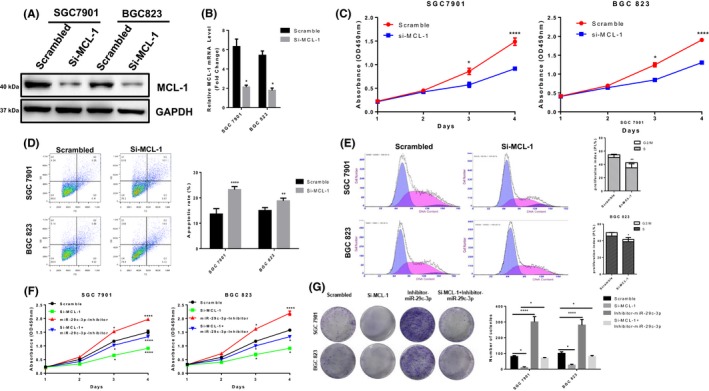
Mcl‐1 expression is up‐regulated in gastric cancer tissues and promotes gastric cancer cell growth. A, The knockdown efficiency of MCL‐1 was determined by Western blotting in SGC‐7901 and BGC‐823 cells. B, MCL‐1 mRNA levels were determined by real‐time quantitative PCR in MCL‐1 knockdown SGC‐7901 and BGC‐823 cells. C, Scrambled siRNA or si‐MCL‐1 was transfected into SGC‐7901 and BGC‐823 cells, and cell proliferation ability was measured by CCK8. D, Scrambled siRNA or si‐MCL‐1 was transfected into SGC‐7901 and BGC‐823 cells and used to detect apoptosis rate by flow cytometry. E, Scrambled siRNA or si‐MCL‐1 was transfected into SGC‐7901 and BGC‐823 cells, and cell cycle was analysed by flow cytometry. F, The proliferation ability of the cells was determined by CCK8 after co‐transfection of si‐MCL‐1, miR‐29c‐3p inhibitor or scrambled siRNA in SGC‐7901 and BGC‐823 cells. G, The proliferation ability of the cells was determined by colony formation assay after co‐transfection of si‐MCL‐1, miR‐29c‐3p inhibitor or scrambled siRNA in SGC‐7901 and BGC‐823 cells. Values represent the mean ± SEM of three independent experiments. *P < .05, **P < .01, ***P < .001

To investigate the potential role of MCL‐1 in GC, we analysed its expression in GC and normal tissues. Similarly, immunohistochemical staining of human GC tissues showed a significant increase in MCL‐1 protein in GC tissues (Figure S2A). Interestingly, Kaplan‐Meier analysis found that higher MCL‐1 expression was significantly associated with shorter overall survival in patients with gastric cancer (Figure S2B). Then, gastric cancer cell lines SGC‐7901 and BGC‐823 were transfected with MCL‐1 siRNA to knockdown their expression, which was confirmed by qRT‐PCR and Western blot (Figure 7A,B). Simultaneously, CCK8 and colony formation assay incorporation assays showed knockdown of MCL‐1 expression significantly reduced cell growth viability and colony formation (Figure 7C,G). In addition, cycle arrest and apoptotic cell rates of GC cells SGC‐7901 and BGC‐823 transfected with si‐MCL‐1 or scrambled siRNA were analysed by flow cytometry (Figure 7D,7). Western blot analysis showed that the expression of cycle‐regulated proteins such as cyclin D1 and CDK2 was significantly decreased in MCL‐1 knockdown SGC‐7901 and BGC‐823 cells, and the expression of apoptosis‐related proteins such as cleaved PARP and cleaved caspase‐3 was significantly increased in these cells (Figure S2C). In addition, knockdown of MCL‐1 inhibits tumour growth in GC cells in *vivo* (Figure S2D,E). Immunofluorescence of Ki‐67 and terminal deoxynucleotidyl transferase‐mediated dUTP‐fluorescein nick end labeling (TUNEL) staining were used in xenograft tissues. The results showed that the knockdown of MCL‐1 reduced Ki‐67–positive cells while increasing the proportion of apoptotic cells. (Figure S2F) Furthermore, proliferation and colony formation were significantly promoted in GC cell lines SGC‐7901 and BGC‐823 transfected with miR‐29c‐3p inhibitors, but this effect was significantly reversed by co‐transfection with MCL‐1–targeted siRNA (Figure 7F,G). Taken together, these data suggest that MCL‐1 promotes GC growth.

## 4 DISCUSSION

Increasing evidences revealed that non‐coding RNAs, particularly lncRNAs and miRNAs, have considerable potential values in improving the diagnostic and therapeutic of GC.^25^, ^26^ Researchers have found a large number of lncRNAs in GC development, such as lncRNA HOXC‐AS3,^27^ LINC01234^4^ and lncRNA‐KRTAP5‐AS1.^28^ Aberrantly expressed lncRNAs are involved in various malignant cytological behaviours of GC cells. Our previously study showed a comprehensive analysis of a novel dysregulated lncRNA‐related ceRNA network in gastric cancer revealing the expression of functional lncRNA in GC.^24^ In the present study, we first discovered the function of lncRNA MYOSLID in GC, which is significantly up‐regulated in GC tissues and cell lines. The high expression of lncRNA MYOSLID is related to AJCC staging, tumour size and depth of invasion. In addition, the high expression of lncRNA MYOSLID expression is associated with a shorter overall survival time in patients with GC. In *vitro* and in *vivo* experiments demonstrated that after knockdown of lncRNA MYOSLID, cell proliferation and tumour growth were significantly inhibited and apoptosis was significantly increased, while overexpression of lncRNA MYOSLID promoted cell proliferation. These findings indicated that lncRNA MYOSLID plays a carcinogenic role in gastric tumorigenesis and can be considered as a potential prognostic indicator of GC.

Meanwhile, a large number of reports have demonstrated that there is a novel and extensive network of interactions involving ceRNAs in the biological function of non‐coding RNAs, in which lncRNA or circRNA can regulate gene expression by competitively binding to miRNAs with mRNA.^29^, ^30^, ^31^ Such as, LINC01234 promotes GC cell proliferation and inhibits cell apoptosis by ceRNA as miR‐204‐5p^4^; lncRNA‐KRTAP5‐AS1 promotes invasion and metastasis of GC cells by ceRNA as miR‐596^28^; and lncRNA‐HOXA11‐AS promotes the proliferation and metastasis ability of GC cells by ceRNA as miR‐1297.^32^ In this study, we found that lncRNA MYOSLID is mainly localized in the cytoplasm by in situ hybridization and interacts with Ago2 in GC cells, suggesting that lncRNA MYOSLID may act as an endogenous miRNA sponge. Then, bioinformatics analysis and luciferase reporter analysis revealed that miR‐29c‐3p is a new target for lncRNA MYOSLID. By analysing TCGA data, it was found that miR‐29c‐3p is down‐regulated in human GC and acts as a tumour suppressor. There is evidence that the expression of miR‐29c‐3p in GC is lower than that in adjacent tissues, and it can significantly inhibit the proliferation of GC cells by down‐regulating the expression of ITGB1.^33^ In addition, it has been reported that miR‐29c is significantly down‐regulated in colon cancer.^34^ In this study, we also found that miR‐29c‐3p was significantly down‐regulated in GC, and we found that increased expression of miR‐29c‐3p inhibited GC cell proliferation and induced apoptosis. At the same time, our results reveal that lncRNA MYOSLID plays an important role in GC cells by sponging miR‐29c‐3p during tumorigenesis and progression.

In general, lncRNA acts primarily by inhibiting miRNAs to affect downstream miRNA targets in the mechanism of the competing endogenous RNAs of lncRNA. Therefore, miRNA targets are an important part of the ceRNA network.^35^, ^36^ Next, we used three online prediction databases and found that MCL‐1 is one of the potential miR‐29c‐3p targets not reported in GC. Meanwhile, to elucidate that miR‐29c‐3p directly targets MCL‐1, we performed a luciferase reporter assay and confirmed that the 3'UTR region of MCL‐1 mRNA is the target site for miR‐29c‐3p. In addition, overexpression of miR‐29c‐3p in GC cells significantly reduced the protein level of MCL‐1. MCL‐1 is a unique anti‐apoptotic BCL‐2 family member that is overexpressed in many tumour types.^37^ For example, Chen G et al reported that targeting MCL‐1 enhanced the sensitivity of DNA replication stress to cancer treatment.^38^ In addition, Zhan Z et al also reported that MCL‐1 can increase the anti‐apoptotic ability of GC cells, thereby promoting the proliferation of GC cells.^39^ In our study, we found that MCL‐1 was significantly up‐regulated in GC tissues compared with normal samples. At the same time, Kaplan‐Meier analysis found that higher MCL‐1 expression was significantly associated with poor overall survival in patients with GC. Then, we found that knockdown of MCL‐1 expression significantly inhibited GC cell proliferation and induced apoptosis. Furthermore, by co‐transfection of MCL‐1 siRNA with the miR‐29c‐3p inhibitor, we found that the function of the miR‐29c‐3p inhibitor can be reversed by MCL‐1 knockdown. These results indicated that miR‐29c‐3p inhibits GC cell proliferation depending on inhibition of MCL‐1 expression.

In conclusion, we report a novel gastric cancer‐associated lncRNA MYOSLID and first discovered that lncRNA MYOSLID is a carcinogenic lncRNA that promotes cell proliferation and inhibits apoptosis in human GC via the miR‐29c‐3p‐MCL‐1 axis. This study provided better understanding of lncRNA‐miRNA‐mRNA ceRNA network in the development of GC. lncRNA MYOSLID may be a potential important target for the diagnosis and treatment of GC.

## CONFLICTS OF INTEREST

The authors declare that they have no competing interests.

## AUTHOR CONTRIBUTIONS

NW, MZJ, YC and ZYW contributed equally. XX, BX and HML designed the study. NW, MZJ, YC and ZYW performed the in *vitro* experiments. YYH, HL and JYC performed the in *vivo* experiments. NW and MZJ analysed the data and wrote the manuscript. YC and ZYW evaluated the histological features. XX and HML supervised the study.
