# Supplementary figures and images for: Long non‐coding RNA MYOSLID functions as a competing endogenous RNA to regulate MCL‐1 expression by sponging miR‐29c‐3p in gastric cancer
Source: Cell Prolif. 2019 Sep 9;52(6):e12678. doi: 10.1111/cpr.12678 (PMC6869334; doi:10.1111/cpr.12678)

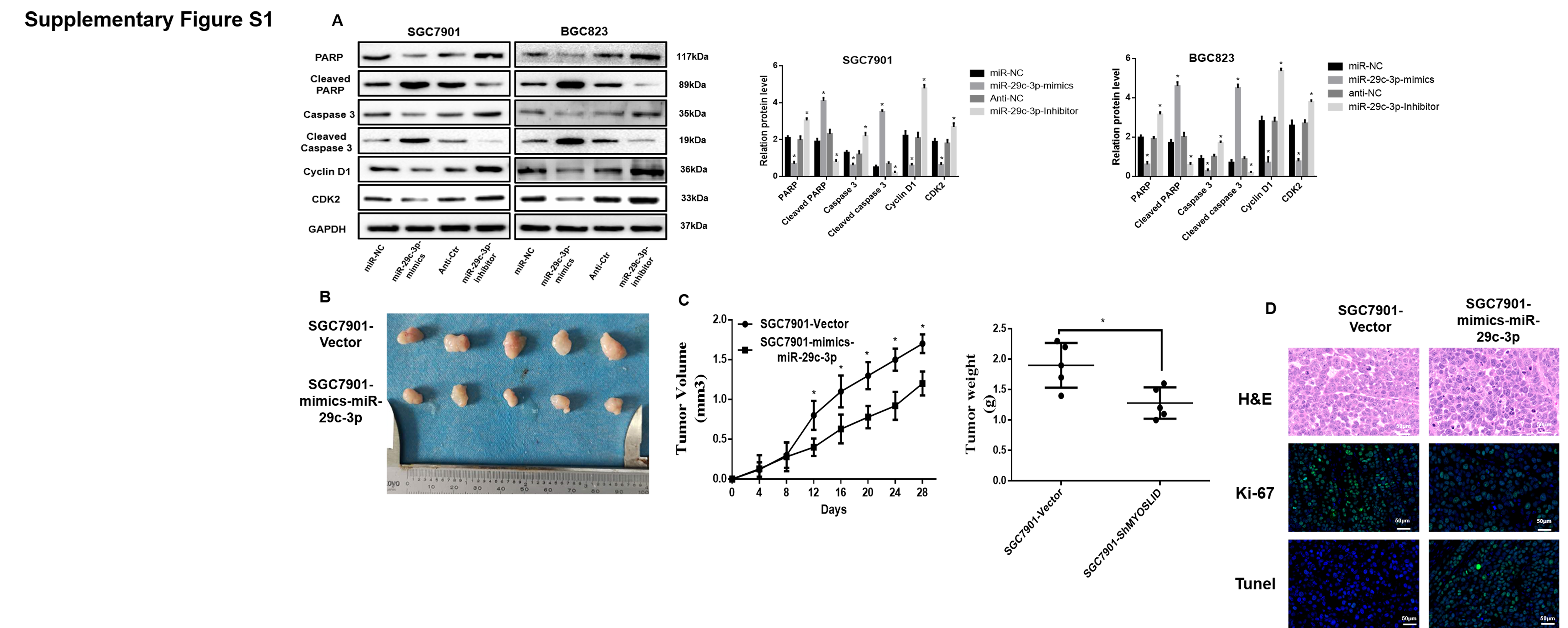

Supplement: Supplementary file 1 [file CPR-52-e12678-s001.tif]

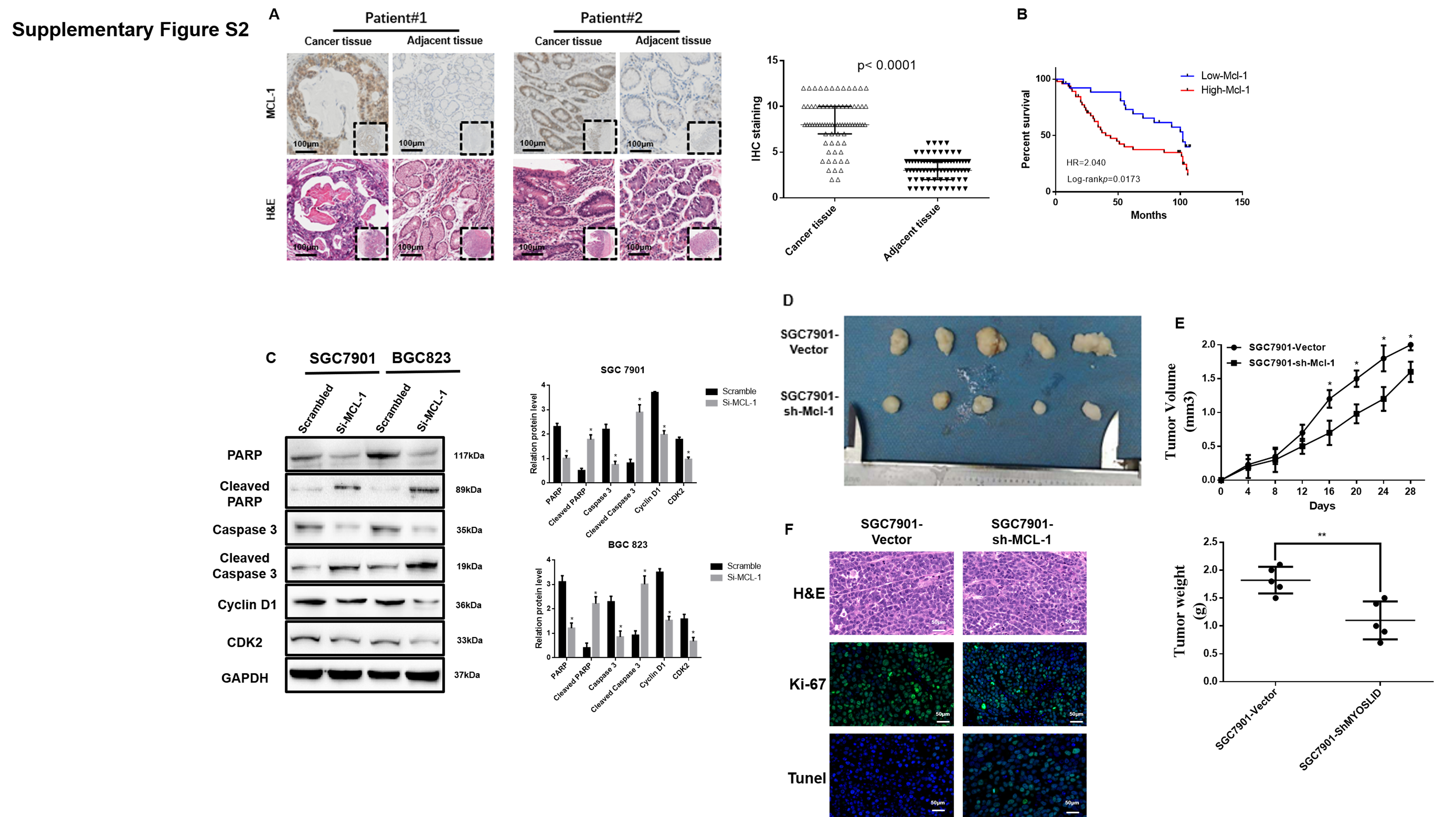

Supplement: Supplementary file 2 [file CPR-52-e12678-s002.tif]
