# Supplementary material for: Long non‐coding RNA MYOSLID functions as a competing endogenous RNA to regulate MCL‐1 expression by sponging miR‐29c‐3p in gastric cancer
Source: Cell Prolif. 2019 Sep 9;52(6):e12678. doi: 10.1111/cpr.12678 (PMC6869334; doi:10.1111/cpr.12678)
Supplement: Supplementary file 3 [file CPR-52-e12678-s003.docx]

**Table S1: MYOSLID levels and clinicopathological features**

**in 75 GC patients**

| **Characteristics** | **Total** | **MYOSLID** | | ***p*** |
| --- | --- | --- | --- | --- |
|  |  | **Low** | **High** |  |
| **Gender** |  |  |  |  |
| **Female** | **26** | **12** | **14** | **1.000** |
| **Male** | **49** | **22** | **27** |  |
| **Age (years)** |  |  |  |  |
| **＞60** | **46** | **26** | **20** | **0.018** |
| **≤60** | **29** | **8** | **21** |  |
| **Tumor size(cm)** |  |  |  |  |
| **＜10** | **12** | **11** | **1** | **0.001** |
| **≥10** | **63** | **23** | **40** |  |
| **Invasion depth** |  |  |  |  |
| **Without Infiltration into Serous layer** | **64** | **33** | **31** | **0.010** |
| **Infiltration into Serous layer** | **11** | **1** | **10** |  |
| **AJCC stage** |  |  |  |  |
| **I\II** | **24** | **18** | **6** | **0.001** |
| **III\IV** | **51** | **16** | **35** |  |
