# Supplementary material for: Long non‐coding RNA MYOSLID functions as a competing endogenous RNA to regulate MCL‐1 expression by sponging miR‐29c‐3p in gastric cancer
Source: Cell Prolif. 2019 Sep 9;52(6):e12678. doi: 10.1111/cpr.12678 (PMC6869334; doi:10.1111/cpr.12678)
Supplement: Supplementary file 4 [file CPR-52-e12678-s004.docx]

**Table S2: The list and sequences of primers used for qRT-PCR**

| **Primer** | **Sequence (5’-3’)** |
| --- | --- |
| MYOSLID-Forward | ACAGGGAGCCAGGACACC |
| MYOSLID-Reverse | GGAACCAGCACCAGGAACC |
| MiR-29c-3p-Forward | GAAAGCCACCACGATGCAACAGACAAATTCTGA |
| MiR-29c-3p -Reverse | TCTGTTGCATCGTGGTGGCTTTCATACTATATC |
| MCL-1-Forward | TGCTTCGGAAACTGGACATC |
| MCL-1-Reverse | TAGCCACAAAGGCACCAAAAG |
| GAPDH-Forward | GAGTCAACGGATTTGGTCGT |
| GAPDH-Reverse | TTGATTTTGGAGGGATCTCG |
| Si-MYOSLID#1 | GGAGAAUGAACUUCUUAAAGCUGAA |
| Si- MYOSLID#2 | GAGCCACCUUGCUCUAGGAUGUGCC |
| Si- MYOSLID#3 | GGAAGAAGCUGUUGAAUCUCUGUAG |
| Sh- MYOSLID | GAGCCACCUUGCUCUAGGAUGUGCC |
| MiR-29c-3p-mimics |  |
| MiR-29c-3p-inhibitor |  |
| Si-MCL-1 |  |

**MYOSLID- sequence:**

GGTTTTCCCAGGGCATGGAAAGGACAGGGCTCCCAGTGGAGATATCATGTAGCTATAGGGCCTAGAAGTATTTTTTCAGTAGATGAAATTTAGAAATAATTTGAATATTTTATGCTGGGGAGGTAGCTTGTGGAGTGAAATGGTCGGGTAAAGGAAACACTGAGTTCAAGTGCTTCTTTATAAAGGACACTTAACTGATCTAAATATTTGTCAAGCTGAATCGCTACATCCACTCTCACCTGCCTGAAATCAATATGCTCAAGTCAAACGCATTTGCCTGCATCTGAATGTGGAGAATGAACTTCTTAAAGCTGAACTGGCAGCTGGAAGGGGAGAGAGAAGAGCTGGACAGGGAGCCAGGACACCGGAGTTCTTTTCCACTGTGGTGGGATCTGGAAGAAGCTGTTGAATCTCTGTAGAGTTAGCTTTTAAATGCTGTGAGTCTTTGGAAAGCTGTGCGAATAACTGCTAAACACCCAGGACAGCTTATGCATTTTTTGAAAAAGGTTCCTGGTGCTGGTTCCAGCAGAATAGACACAGAGATGCATGGCCTAACTCCCACTCCCTCTTCAGGACTGAGCCACGCAGCCCCCCATCTGCCAGAAGTGTTAGCTGTTGACGGCTCACAGCTGAGTCCCTCTCCAGGCATTGCTGAAAAGAGCCACCTTGCTCTAGGATGTGCCCCCACCCTGGGGGCAGCATACATTCCAAGTGATTAGTCCATAGTGTGGTACAAAGGTTGTACCCATCTTGCCTGAATTCAGGATCTCTTGGAAAACCACCCCAGCTCTGCAGCTCCTGGTGGGGTGCTGAGGCCTCTGCTGCAGCTGCATCGCAGTTTACTTTCCCCTCTGCCTAGTCCTGCTGCCTTCACTCCCCAACAGATCACCCGCTCTGAACATGCCATCTACTGTGGAAATCTTCAATCTTTAATTAATTTTTCCTGTTACAGGAAGATTAAGTCTAAAAGGGTATCTCCCCTAGTTGATCCAGAACTAAATGACATTGTTTTCAGTACTTCCAGTAACTTGCTTACACTCTTACTTCTAAAGCATTGAATGTATCATCTTATGTGCTTGTCACCCCCAGCTTGTGTCATAGGAGAAAGTATACGGAGATCAGGTACAACTTCAGTTCAACAGCTTATGAGCTATTGGATCAGTCTCCTTATTAATCTCTCAGAGCCTCAATTTCCTTGTTTAAAATGGGGCAATACCTACCTTAAGGAATTTCTTGAGGATCAAAAGAAACCCTAGTAACAAAGCCTTGAACCATGTCTGACTTATAGTATATATTCTAACTGCA
