# Supplementary material for: Long non‐coding RNA MYOSLID functions as a competing endogenous RNA to regulate MCL‐1 expression by sponging miR‐29c‐3p in gastric cancer
Source: Cell Prolif. 2019 Sep 9;52(6):e12678. doi: 10.1111/cpr.12678 (PMC6869334; doi:10.1111/cpr.12678)
Supplement: Supplementary file 5 [file CPR-52-e12678-s005.docx]

**Table S3: The list of primary antibodies used**

| **Target** | **Usage** |  | **Source** | **Catalog number** | **Dilution** |
| --- | --- | --- | --- | --- | --- |
| MCL-1 | WB |  | CST | 94296 | 1:1000 |
| MCL-1 | IHC |  | CST | 94296 | 1:200 |
| PARP | WB |  | CST | 9532 | 1:1000 |
| Cleaved-PARP | WB |  | CST | 5625 | 1:1000 |
| Caspase-3 | WB |  | CST | 9662 | 1:1000 |
| Cleaved-Caspase3 | WB |  | CST | 9664 | 1:1000 |
| Cyclin D1 | WB |  | CST | 2978 | 1:1000 |
| CDK2 | WB |  | CST | 2546 | 1:1000 |
| Ki-67 | WB |  | Abcam | ab15580 | 1:100 |
| GAPDH | WB |  | Proteintech | 10494-1-AP | 1:1000 |
