# Supplementary material for: Long non‐coding RNA MYOSLID functions as a competing endogenous RNA to regulate MCL‐1 expression by sponging miR‐29c‐3p in gastric cancer
Source: Cell Prolif. 2019 Sep 9;52(6):e12678. doi: 10.1111/cpr.12678 (PMC6869334; doi:10.1111/cpr.12678)
Supplement: Supplementary file 6 [file CPR-52-e12678-s006.docx]

**Supplementary Experimental Method：**

**Cell transfection**

GC cell lines were transfected with human-targeted double-stranded lncRNA MYOSLID and MCL-1 (purchased from RiboBio, Guangzhou, China), and specific siRNA and scrambled siRNA were cultured with Opti-MEM (Invitrogen, Karlsruhe, Germany). The base was mixed with HiPerFect® transfection reagent (Qiagen, Dusseldorf, Germany) for transfection and cultured according to the manufacturer's instructions. Negative control miRNA, miR-29c-3p mimics and miR-29c-3p inhibitor oligonucleotides (purchased from RiboBio, Guangzhou, China). In all experiments, scrambled siRNA was used as a control. After 48 hours of transfection, the cells were harvested for qRT-PCR or Western blot analysis.

**Cell viability assay**

SGC-7901 and BGC-823 cells, which were transfected with si-MYOSLID (1000 cells/well) for 24 hours, were cultured in 96-well plates and cultured at 37 ° C under a 5% CO_2_ atmosphere. Ten microliters of Cell Counting Kit-8 solution (Dojindo, Kumanoto, Japan) was added to each well according to the manufacturer's instructions and incubated for 2 hours at 37 ° C in a 5% CO_2_ humidified incubator. The spectrometer Varioskan® Flash (Thermo Fisher, Waltham, USA) was used to measure the absorbance at 450 nm. A proliferation curve is drawn in which time is taken as the abscissa and the average absorbance value in each group is taken as the ordinate.

**Colony formation assay**

A total of 500 SGC-7901 and BGC-823 cells transfected with si-MYOSLID were placed in each well of a six-well plate and cultured in appropriate medium containing 10% FBS for about 14 days. After 14 days, it was fixed with absolute ethanol, stained with 0.1% crystal violet (Beyotime Biotechnology, Shanghai, China), and colony formation was determined by counting the number of stained colonies. Colony formation assays were performed to monitor GC cell clonality.

**Ethynyldeoxyuridine (EdU) analysis**

The SGC-7901 and BGC-823 cells transfected with si-MYOSLID were evaluated for proliferating cells using a 5-ethynyl-2-deoxyuridine (EdU) labeling/detection kit (Ribobio, Guangzhou, China) according to the manufacturer's protocol. The cells were cultured in 96-well plates. 5 x10^3^ cells per well, then 50 μM EdU labeling medium was added to a 96-well plate and incubated for 3 hours at 37°C, 5%CO_2_. 4% paraformaldehyde and 0.5% Triton X-100, cells were stained with anti-EdU working solution, DAPI was used to label the nuclei, and analyzed by fluorescence microscopy.

**Luciferase reporter assay**

For the dual luciferase reporter assay, a complementary DNA fragment containing the wild type or mutant lncRNA MYOSLID fragment and the 3' untranslated region (UTR) of MCL-1 was subcloned into the pGL3-Baisc luciferase reporter vector (Promega) Downstream of the luciferase gene. When cells reached 60% confluence in 24-well plates, they were transfected with X-tremegene HP (Roche). The firefly luciferase reporter construct (0.1μg) and the Renilla luciferase construct (0.02μg) were co-transfected in wells. In addition, luciferase activity was measured 48 hours after transfection using a dual luciferase reporter assay system (Promega) according to the manufacturer's instructions.

**Immunohistochemical (IHC) staining:**

Mcl-1 (D2W9E) Rabbit mAb #94296 was used for IHC analyses, with tissue staining performed as previously published. On each slide, both the IHC staining score of positive cells and the intensity of the positive cells were calculated using the semiquantitative scoring method[[1](#_ENREF_1)]. The immunostaining intensity was evaluated as previously described[[2](#_ENREF_2)].

1. **B X, M J, Y C, W W, D C, X L, Z Z, D Z, D F, Y N, F S, K W, J L.** Gasdermin D plays a key role as a pyroptosis executor of non-alcoholic steatohepatitis in humans and mice. *Journal of hepatology*. 2018; 68: 773-82.

2. **M J, B X, X L, Y S, Y C, W W, D C, N W, S H, S Z, M L, K W, X Y, J L, Y N, D F.** O-GlcNAcylation promotes colorectal cancer metastasis via the miR-101-O-GlcNAc/EZH2 regulatory feedback circuit. *Oncogene*. 2019; 38: 301-16.
